# Diagnostic and surveillance testing capability for mpox in the EU/EEA, September 2024

**DOI:** 10.2807/1560-7917.ES.2024.29.42.2400632

**Published:** 2024-10-17

**Authors:** Nina Lagerqvist, Jessica Beser, Tamás Bakonyi, Céline M Gossner, Daniel Palm

**Affiliations:** 1 European Centre for Disease Prevention and Control (ECDC), Stockholm, Sweden

**Keywords:** mpox, laboratory capability, MPXV, diagnostic testing, clade differentiation, EU/EEA, PCR, whole genome sequencing

## Abstract

In response to the increasing number of mpox cases caused by monkeypox virus (MPXV) clade I in the African continent and the first reported travel-related clade Ib case of mpox in EU/EEA, the European Centre for Disease Prevention and Control surveyed national capability for detection and characterisation of MPXV in the EU/EEA. The results showed high level of capability for case confirmation by PCR, alongside molecular typing methods for identification of MPXV clades and/or clade I subclades within the EU/EEA.

On 14 August 2024, the World Health Organization (WHO) declared a Public Health Emergency of International Concern (PHEIC) because of an increased number of mpox cases in the Democratic Republic of the Congo (DRC) and neighbouring countries [1]. Both monkeypox virus (MPXV) clades I and II circulate in the African continent. However, since the end of 2023, MPXV clade I has upsurged in Central and Eastern Africa [2]. Occasional cases among travellers returning from this region were expected, and in August 2024, one travel-related case of clade Ib was reported in the European Union/European Economic Area (EU/EEA) and one in Thailand [3,4]. In September 2024, the European Centre for Disease Prevention and Control (ECDC) conducted a survey to rapidly ascertain the laboratory capabilities of EU/EEA countries to diagnose, characterise and report mpox cases.

## Survey on mpox laboratory testing capabilities in the EU/EEA

The survey was distributed to the 30 EU/EEA countries and covered the following five areas: (i) capability to diagnose mpox; (ii) capability to type MPXV to clade/subclade level; (iii) capability for MPXV whole genome sequencing (WGS); (iv) aspects of reporting of laboratory data to surveillance systems; and (v) needs for mpox laboratory support.

The survey was sent by email on 3 September 2024 to the ECDC National Microbiology Focal Points of the EU/EEA countries with a deadline of 6 September 2024. The National Microbiology Focal Points are ECDC official contact points nominated by the EU/EEA countries with specific knowledge of the national microbiology systems [5]. Reminders were sent on 9 and 11 September; data were collected until 13 September 2024 and were validated by the survey respondents 18–19 September 2024.

The survey had a response rate of 100%, capturing data from all 30 EU/EEA countries.

## Capability of EU/EEA countries to diagnose mpox and characterise MPXV

All EU/EEA countries reported capability to diagnose mpox using PCR, which is the preferred laboratory diagnostic method [6].

**Figure 1:**
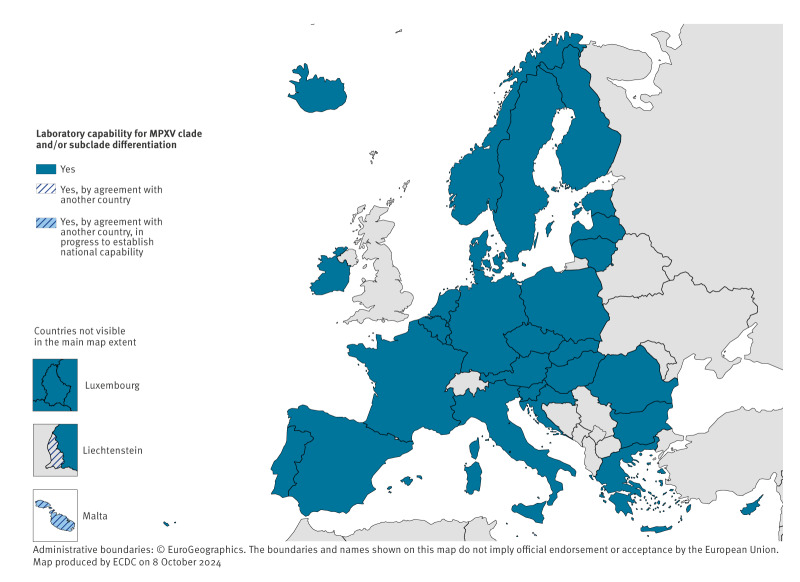
MPXV clade/subclade differentiation capability in the 30 EU/EEA countries, September 2024

Twenty-eight countries reported capability to differentiate between MPXV clades (I and II) and/or clade I subclades (Ia and Ib) using either specific PCR assays, sequencing or both techniques. The remaining two countries reported access to this capability through an agreement with another country (Figure 1).

EU/EEA: European Union/European Economic Area; MPXV: monkeypox virus.

Among the countries reporting national capability to differentiate between MPXV clades/subclades, two countries reported having specific PCR tests, six reported having sequencing approaches available and 20 countries reported having both specific PCR tests and sequencing available for this purpose.

Among the 22 countries reporting having specific PCR tests for clade/subclade differentiation, 12 reported having PCR tests available for distinction between both clades and clade I subclades, six reported having assays for clade differentiation only and two countries reported having assays for differentiating clade I subclades only. Two countries did not answer the question.

**Figure 2:**
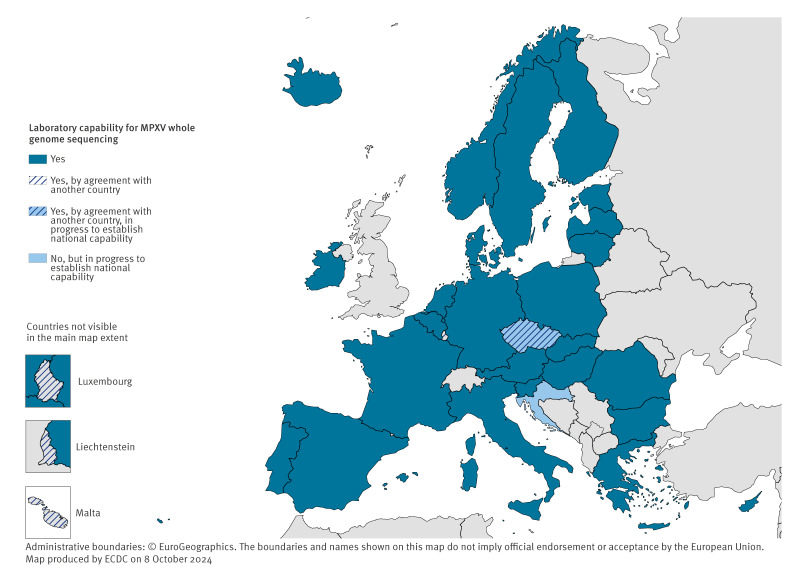
MPXV whole genome sequencing capability in the 30 EU/EEA countries, September 2024

Twenty-five countries reported national capability for WGS, covering at least 90% of the MPXV genome for a typical sample, and four countries reported access to this capability through an agreement with another country. One country reported lacking both national and outsourced capability of MPXV WGS but were planning to implement this at national level in the coming months (Figure 2).

EU/EEA: European Union/European Economic Area; MPXV: monkeypox virus.

Twenty countries with national or outsourced WGS capability reported that they deposit MPXV sequences in the public domain through open-access databases such as European Nucleotide Archive, Sequence Read Archive or GenBank, or to GISAID, which has specific requirements on registration and data use. Of the nine countries not depositing sequences to these databases, six indicated that this was either due to the absence of cases or because sequencing was not routinely performed at the time of the survey, and three countries did not provide additional information to this question.

## Reporting of MPXV clade/subclade

Twenty-eight countries reported they were able to collect information about MPXV clades and link this data with epidemiological information at the national level.

Fourteen countries indicated that they were reporting clade or subclade information to the EU surveillance system at the time of the survey. Nine of the 16 countries not currently reporting this information to the EU surveillance system provided additional information. Of these, three attributed the lack of reporting to having no cases this year, four indicated plans to begin reporting in the near future and two countries stated other reasons.

## Discussion

The outbreak declared in 2022 and caused by MPXV clade II (subclade IIb) spread rapidly across multiple previously non-endemic countries worldwide, with all EU/EEA countries (except Liechtenstein) eventually reporting locally acquired cases [7]. The outbreak was driven by human-to-human MPXV transmission via close contact with infected individuals, with a majority of cases reported among men who have sex with men presenting with mainly mild clinical manifestations [7]. The WHO declared a PHEIC in July 2022 that was later lifted in May 2023 following a sustained decline in cases globally [8]. The outbreak of MPXV clade II continues to this day, including in the EU/EEA, albeit with a lower number of cases [9].

While infections with MPXV clade I are known to sporadically occur in the DRC [10], the current outbreaks of clade I are more widespread than previously described outbreaks in DRC, with recent geographical expansion to other African countries [11]. The observed increase in clade I infections are due to MPXV subclades Ia and Ib, with subclade Ib first detected in DRC in October 2023 [12]. All age groups have been affected and both human-to-human transmission through close contact (e.g. sexual transmission, household transmission) and zoonotic transmission have been reported [11].

Historically, clade I was reported to cause more severe illness than clade II [13], but more comprehensive epidemiological data are required to determine potential differences between clades in disease severity, main transmission routes and transmissibility [14]. Early detection and characterisation of imported cases of MPXV clade I in the EU/EEA are imperative to inform public health actions [15]. The results of this mapping demonstrate a robust capability for case confirmation by PCR, alongside molecular typing methods for identification of clades and subclades across EU/EEA countries. The limitations of this exercise include that the survey did not capture aspects on specific assays used in laboratories across EU/EEA countries, or capacities to handle higher volumes of testing, should this be needed.

Mpox case confirmation for reporting to EU level surveillance follows the WHO case definition [16] and includes detection of MPXV by PCR and/or sequencing [17]. Since 20 August 2024, The European Surveillance System (TESSy) reporting protocol defines categories to report MPXV clade and subclade data [17]. 

## Conclusion

Public health authorities in EU/EEA countries should ensure early detection and investigation of mpox clade I cases and complete data reporting to ECDC for EU level analysis. Countries are also encouraged to sequence and share data from all positive mpox specimens to increase our – much needed – understanding of viral evolution, transmission routes and patterns of spread.
